# Efficiency of succinylated gelatin and amino acid infusions for kidney uptake reduction of radiolabeled αvβ6-integrin targeting peptides: considerations on clinical safety profiles

**DOI:** 10.1007/s00259-024-06738-2

**Published:** 2024-05-08

**Authors:** Stefan Stangl, Nghia Trong Nguyen, Julia Brosch-Lenz, Jakub Šimeček, Wolfgang A. Weber, Susanne Kossatz, Johannes Notni

**Affiliations:** 1 Department of Nuclear Medicine, University Hospital Klinikum Rechts Der Isar, School of Medicine and Health, Technical University of Munich, Munich, Germany; 2 Central Institute for Translational Cancer Research (TranslaTUM), School of Medicine and Health, Technical University of Munich, Munich, Germany; 3 TRIMT GmbH, Radeberg, Germany; 4 Department of Chemistry, School of Natural Sciences, Technical University of Munich, Munich, Germany; 5 Institute of Pathology, School of Medicine and Health, Technische Universität München, München, Germany

**Keywords:** Kidney protection, Peptide receptor radionuclide therapy, Succinylated gelatin, Arginine, Lysine

## Abstract

**Purpose:**

^68^Ga-Trivehexin is an investigational PET radiopharmaceutical (NCT05799274) targeting αvβ6-integrin for PET imaging of carcinomas. ^177^Lu-D0301 is a structurally related therapeutic peptide tetramer. However, it showed considerable kidney uptake in rodents, impeding clinical applicability. We therefore evaluated the impact of different kidney protection strategies on the biodistribution of both agents in normal and tumor-bearing mice.

**Methods:**

Ex-vivo biodistribution of ^68^Ga-Trivehexin (90 min p.i.) and ^177^Lu-D0301 (90 min and 24 h p.i.) was determined in healthy C57BL/6N and H2009 (human lung adenocarcinoma) xenografted CB17-SCID mice without and with co-infusion of 100 µL of solutions containing 2.5% arginine + 2.5% lysine (Arg/Lys), 4% succinylated gelatin (gelofusine, gelo), or combinations thereof. Arg/Lys was injected either i.p. 30 min before and after the radiopharmaceutical, or i.v. 2 min before the radiopharmaceutical. Gelo was administered either i.v. 2 min prior activity, or pre-mixed and injected together with the radiopharmaceutical (*n* = 5 per group). C57BL/6N mice were furthermore imaged by PET (90 min p.i.) and SPECT (24 h p.i.).

**Results:**

Kidney uptake of ^68^Ga-Trivehexin in C57BL/6N mice was reduced by 15% (Arg/Lys i.p.), 25% (Arg/Lys i.v.), and 70% (gelo i.v.), 90 min p.i., relative to control. ^177^Lu-D0301 kidney uptake was reduced by 2% (Arg/Lys i.p.), 41% (Arg/Lys i.v.), 61% (gelo i.v.) and 66% (gelo + Arg/Lys i.v.) 24 h p.i., compared to control. Combination of Arg/Lys and gelo provided no substantial benefit. Gelo furthermore reduced kidney uptake of ^177^Lu-D0301 by 76% (90 min p.i.) and 85% (24 h p.i.) in H2009 bearing SCID mice. Since tumor uptake was not (90 min p.i.) or only slightly reduced (15%, 24 h p.i.), the tumor/kidney ratio was improved by factors of 3.3 (90 min p.i.) and 2.6 (24 h p.i.). Reduction of kidney uptake was demonstrated by SPECT, which also showed that the remaining activity was located in the cortex.

**Conclusions:**

The kidney uptake of both investigated radiopharmaceuticals was more efficiently reduced by gelofusine (61–85%) than Arg/Lys (25–41%). Gelofusine appears particularly suitable for reducing renal uptake of αvβ6-integrin targeted ^177^Lu-labeled peptide multimers because its application led to approximately three times higher tumor-to-kidney ratios. Since the incidence of severe adverse events (anaphylaxis) with succinylated gelatin products (reportedly 0.0062–0.038%) is comparable to that of gadolinium-based MRI or iodinated CT contrast agents (0.008% and 0.04%, respectively), clinical use of gelofusine during radioligand therapy appears feasible if similar risk management strategies as for contrast agents are applied.

## Introduction

Radioligand therapy (RLT) with particle-emitting radionuclides such as ^177^Lu or ^225^Ac for tumor therapy arguably had the greatest impact on the practice of nuclear medicine in the last ten years [1]. Ideally, such radiopharmaceuticals are selectively accumulated and retained in the tumor tissue while eventually being eliminated from all non-target organs. Particular attention must be paid to the excretory organs, which can be exposed to high radiation levels during the elimination of the radioactive compounds. Renal radiation doses are a specific concern. The mass of a human kidney is only about 150 g (about 0.2% of the total body mass) meaning that potentially toxic radiation doses can be reached if only a small fraction of the injected activity is retained in the kidney parenchyma.

Virtually all radiolabeled small molecules, peptides and lightweight proteins < 60 kDa undergo glomerular filtration, but most are reabsorbed into proximale tubular cells by the megalin/cubilin receptor system to recover amino acids and other essential components from peptides and proteins [2]. Its involvement in the renal reabsorption of ^111^In-labeled octreotide, octreotate, minigastrin, exendin, and neurotensin proves a high degree of promiscuity and suggests that megalin/cubilin blockade is a quite universal approach to achieve a lower renal uptake of radiolabeled peptides [3]. For radiolabeled somatostatin receptor ligands, it has been first shown that the retention of radioactivity in the kidneys can be significantly decreased by co-injection of the cationic amino acids arginine and lysine [4] because they partly inhibit the megalin/cubilin mediated tubular reabsorption [5, 6]. This approach was clinically evaluated about 20 years ago [7], and being considered safe and effective, has become the current clinical standard for RLT using radiolabeled somatostatin analogs [8]. When using the approved radiopharmaceutical Lutathera®, also known as [^177^Lu]Lu-DOTATATE, the risk of kidney damage due to renal retention of radioactivity is routinely mitigated by infusion of a solution containing 2.5% Arg and 2.5% Lys (a total of 25 g of each) from 30 min before the application of the radiopharmaceutical until at least 4 h afterward.

There are several types of blood plasma substitutes that have been, or still are, widely used in emergency medicine and in the perioperative setting, among them solutions of colloids like hydroxyethyl starch (HES, 6% hydroxyethyl starch, MW 130 kDa, in saline; marketed e.g. as Volulyte®), albumin, and 4% succinylated bovine gelatin in saline, marketed as Gelafundin®, Gelofusine®, or Gelaspan® in Europe, or Isoplex® and Volplex® in the US (we herein refer to it as ‘gelofusine’ because of a widespread use of the term in previous literature). The observation that an infusion of succinylated gelatin increased low molecular weight proteinuria by blockade of tubular reabsorption prompted an investigation of plasma expanders for renal protection during RLT [9]. While HES showed no significant effect, gelofusine reduced the renal uptake of ^111^In-DOTATOC by 45 ± 10% at a dose of averagely 12.9 g succinylated gelatin, with no side effects. A comparable effect (up to 50% kidney uptake reduction) was only reported for a large dose of 75 g of Lys [7], which, however, never reached routine use because of the risk of severe hyperkalemia.

Radiolabeled exendins are a classic example of radiopharmaceuticals that have a high potential for theranostic applications due to their tumor-specific expression, but whose clinical application in RLT has not yet been realized due to an unfavorably high renal uptake. Recently, the use of gelofusine in connection with the glucagon-like peptide 1 receptor (GLP-1R) ligand ^111^In-DOTA-exendin-4 resulted in a reduced renal retention by 18% in patients without lowering the tumor uptake [10]. There were large interindividual differences, and for 3 patients, reduction was so strong that these patients would have been eligible for GLP-1R targeted RLT. However, although ^68^Ga-NOTA-exendin-4 can be regarded as quite similar to ^111^In-DOTA-exendin-4, gelofusine reduced the renal uptake of the ^68^Ga-labeled peptide by up to 57% [11].

**Fig. 1:**
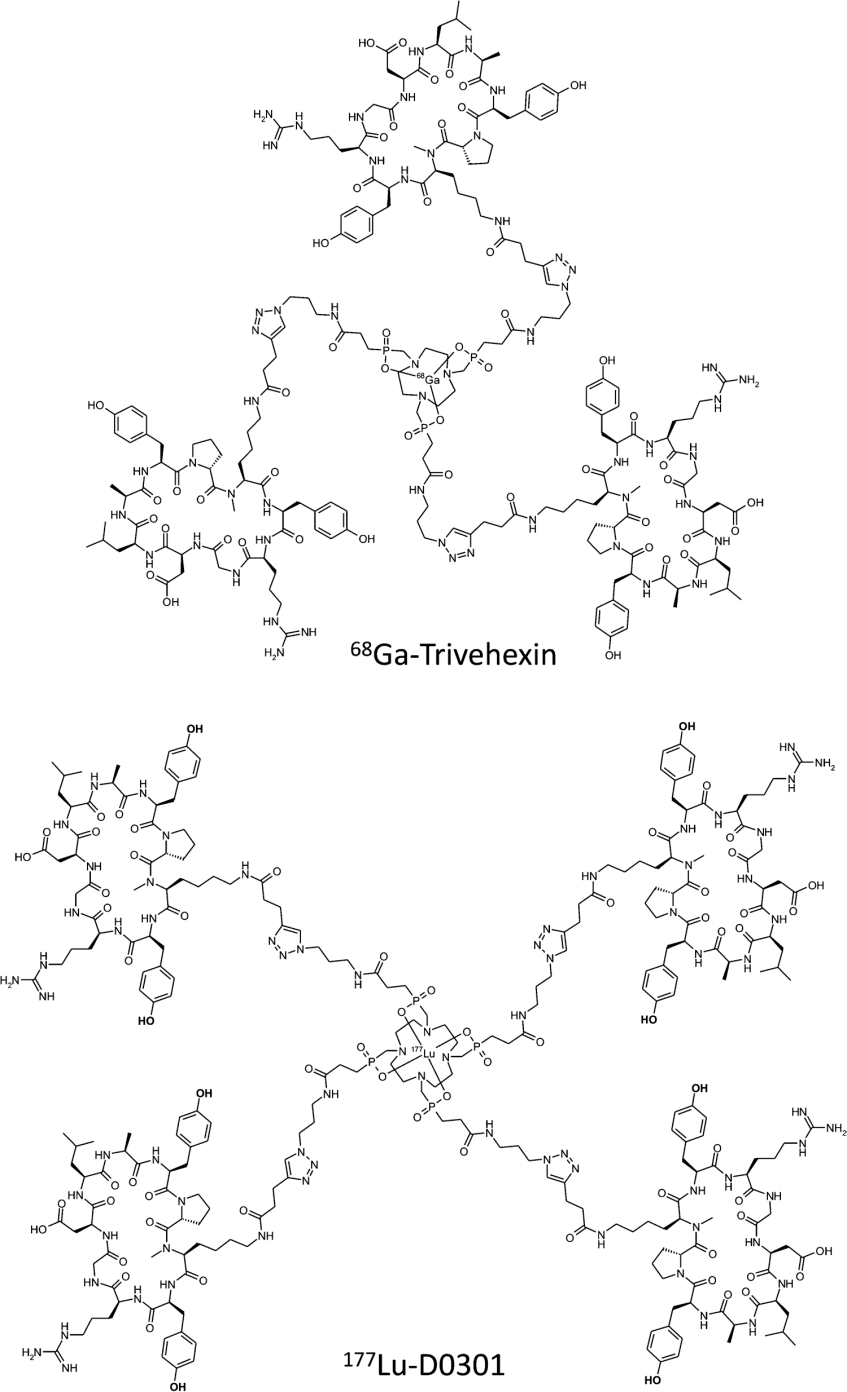
Structures of ^68^Ga-Trivehexin and ^177^Lu-D0301

A high renal retention was also observed for αvβ6-integrin targeted radiopharmaceuticals based on cyclopeptide multimers [12]. We recently introduced a ^68^Ga-labeled trimer of the peptide c[YRGDLAYp(*N*Me)K], referred to as ^68^Ga-Trivehexin (see Fig. 1) [13], which has been successfully applied for PET/CT imaging of pancreatic ductal adenocarcinoma (PDAC) [14] as well as head-and-neck squamous cell carcinoma (HNSCC) [15]. Since αvβ6-integrin is upregulated in various malignant cancers [16], especially in pancreatic ductal adenocarcinoma (PDAC) [17], oral squamous cell carcinoma (OSCC) [18], ovarian [19] and cervical cancer [20], and in non-small cell lung cancer NSCLC [21] as well as its brain metastases [22], the receptor is considered a promising theranostic target [23]. To this end, we recently investigated ^177^Lu-labeled tetramers of c[YRGDLAYp(*N*Me)K], such as the larger Trivehexin congener ^177^Lu-D0301 (see Fig. 1, previously also termed Y8) [24], for application in αvβ6-integrin targeted RLT. However, biodistribution in rodents showed unfavorably high renal uptake. We therefore evaluated kidney protection strategies for our compounds in a preclinical setting.

## Materials and methods

### General

Arg/Lys was prepared by the in-house pharmacy as buffered (pH 7.4) sterile-filtered solution, by dissolving 12.5 g arginine hydrochloride and 12.5 g lysine hydrochloride in water, adjusting the solution to pH 7.4 with 1 M sodium hydroxide, filling to a volume of 500 mL, and sterile filtration. 4% succinylated gelatin was obtained from B.Braun (Melsungen, Germany; brand name in Germany: Gelafundin®) in 500 mL infusion bags. ^68^Ga-Trivehexin [13] and ^177^Lu-D0301 [24] were synthesized as described previously.

### Cell lines and animal models

All animal experiments were approved by the responsible authority (Regierung von Oberbayern) and have been performed in accordance with general animal welfare regulations in Germany and the institutional guidelines for the care and use of animals. Female CB17 severe combined immunodeficiency (SCID) mice and C57BL/6 mice were obtained from Charles River (Sulzfeld, Germany). Keeping of the animals, generation of subcutaneous H2009 tumor xenografts, and ex-vivo biodistribution studies were done according to previously described protocols [25]. Briefly, H2009 cells (*American Type Culture Collection (ATCC)*, Manassas, VA, USA) were cultivated as recommended by the distributor, and were tested regularly to exclude mycoplasma contamination. Tumor xenografts were generated by inoculating 6–8 weeks old female CB17 SCID mice with 5 × 10^6^ cells in Matrigel® (Geltrex™ LDEV-Free Reduced Growth Factor Basement Membrane Matrix, A1413202, Life Technologies, Thermo Fisher Scientific). The mice were used for biodistribution or PET when tumors had grown to a diameter of 8–10 mm (5–6 weeks after inoculation).

### Biodistribution

For biodistribution analysis, animals were injected intravenously with approximately 5–10 MBq ^68^Ga-Trivehexin or 2–3 MBq ^177^Lu-D0301 with or without kidney protection. After 90 min (^68^Ga- and ^177^Lu-labeled compounds) or 24 h (^177^Lu-labeled compounds only), the mice were anesthetized with 2% isoflurane, sacrificed via craniocervical dislocation, and dissected. Organ weights were determined using a Sartorius analytical balance. A Wizard^2^ gamma counter (PerkinElmer) was used to determine radioactivity in each resected organ after calibration with dilutions of the respective radiopharmaceutical.

### PET and SPECT imaging

Selected animals from the biodistribution cohort (*n* = 1–2/group) were subjected to in vivo imaging using a PET/MR 3 T or SPECT/CT scanner (both nanoscan series, Mediso) prior to reaching their endpoint. PET imaging of ^68^Ga-Trivehexin injected mice was performed for 15 min (75–90 min p.i.), preceded by an MR scan for anatomical correlation. SPECT imaging of ^177^Lu-D0301 injected mice was performed for 60 min (23–24 h p.i.) and preceded by a CT scan for anatomical correlation. Reconstruction, image analysis and quantification of PET/MR and SPECT/CT data and image analysis were performed using Nucline and Interview fusion software (both Mediso). MRI sequence parameters: GRE (gradient echo) 3D 0.25 mm iso, NEX: 3 (number of averages), *T*_R_: 20 ms, *T*_E_: 4.0 ms. PET reconstruction parameters: TT3D, It:4, Ss:6, 400–600 keV, 1:3, R:0.0005, M:24.

## Results

### Influence of renal protection on biodistribution in normal mice

We first used non-tumor bearing C57BL/6 mice to screen a variety of application schemes for kidney protection agents in rodents that occurred in the pertinent literature (Table 1). Since the most substantial variation was the injection route (intraperitoneally, i.p., vs. intravenously, i.v.), this aspect was investigated first. The effects of i.v. administration of Arg/Lys and gelofusine were compared using the same injection scheme, i.e., infusions being given 2 min before the radiopharmaceutical. We also investigated whether kidney uptake reduction effects of both agents are cumulative if injected simultaneously. Finally, the feasibility of co-injection of gelofusine with the radiopharmaceutical from a single syringe was tested, because a formulation containing both the tracer and the kidney protection agent could potentially improve clinical workflows and reduce radiolysis due to a larger dilution of the radioactivity.

**Table 1:** Biodistribution experiments performed in C57BL6 mice (*n* = 5/group)

Tracer	Time p.i	Kidney protection scheme	Dose [mg/kg]
^68^Ga-Trivehexin	90 min	none	
		2 × 100 µL Arg/Lys, i.p., 30 min before / after activity	2 × 250
		100 µL Arg/Lys, i.v., 2 min before activity	250
		100 µL gelofusine, i.v., 2 min before activity	200
^177^Lu-D0301	24 h	none	
		2 × 100 µL Arg/Lys, i.p., 30 min before / after activity	2 × 250
		100 µL Arg/Lys, i.v., 2 min before activity	250
		100 µL gelofusine, i.v., 2 min before activity	200
		100 µL Arg/Lys + 100 µL gelofusine, i.v., 2 min before activity	250 + 200
		100 µL gelofusine, mixed with activity	200

**Fig. 2:**
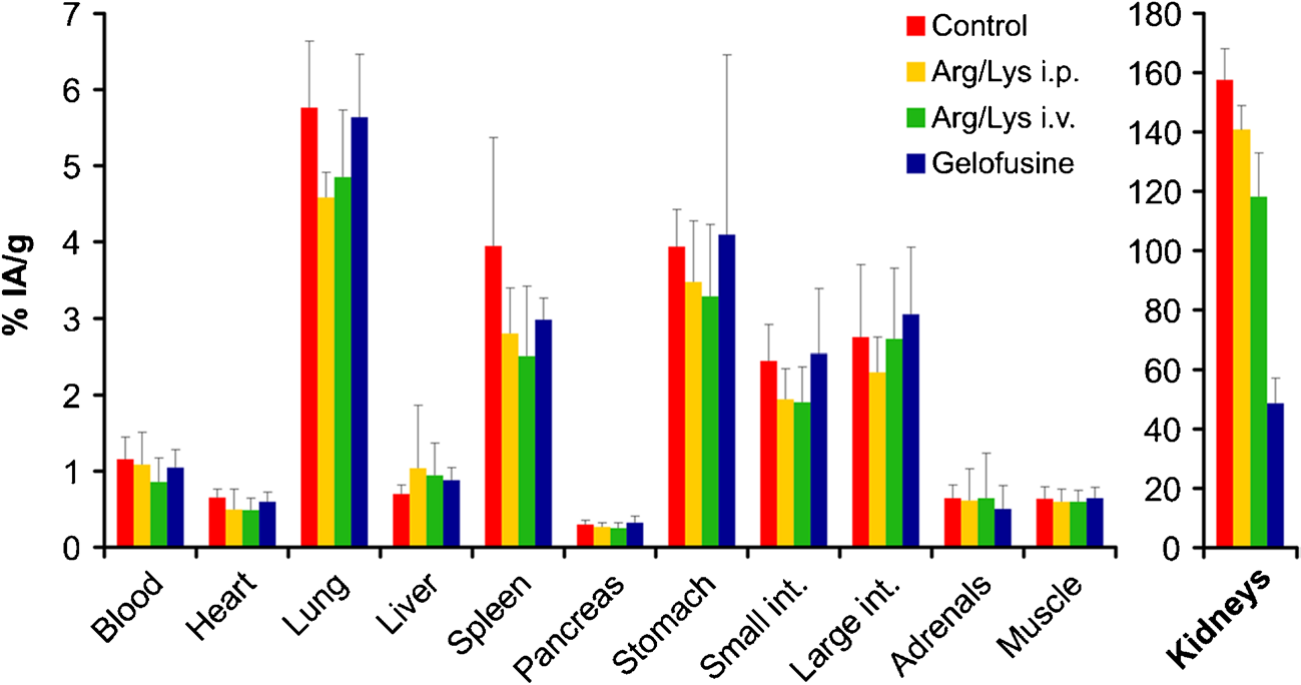
Biodistribution of ^68^Ga-Trivehexin in C57BL/6 non-tumor bearing mice (90 min p.i.). Control: no kidney protection. Arg/Lys i.p.: 2 × 100 µL Arg/Lys, i.p., 30 min before/after activity. Arg/Lys i.v.: 100 µL Arg/Lys, i.v., 2 min before activity. Gelofusine: 100 µL gelofusine, i.v., 2 min before activity. Data are displayed as averages ± SD of *n* = 5 per group

**Fig. 3:**
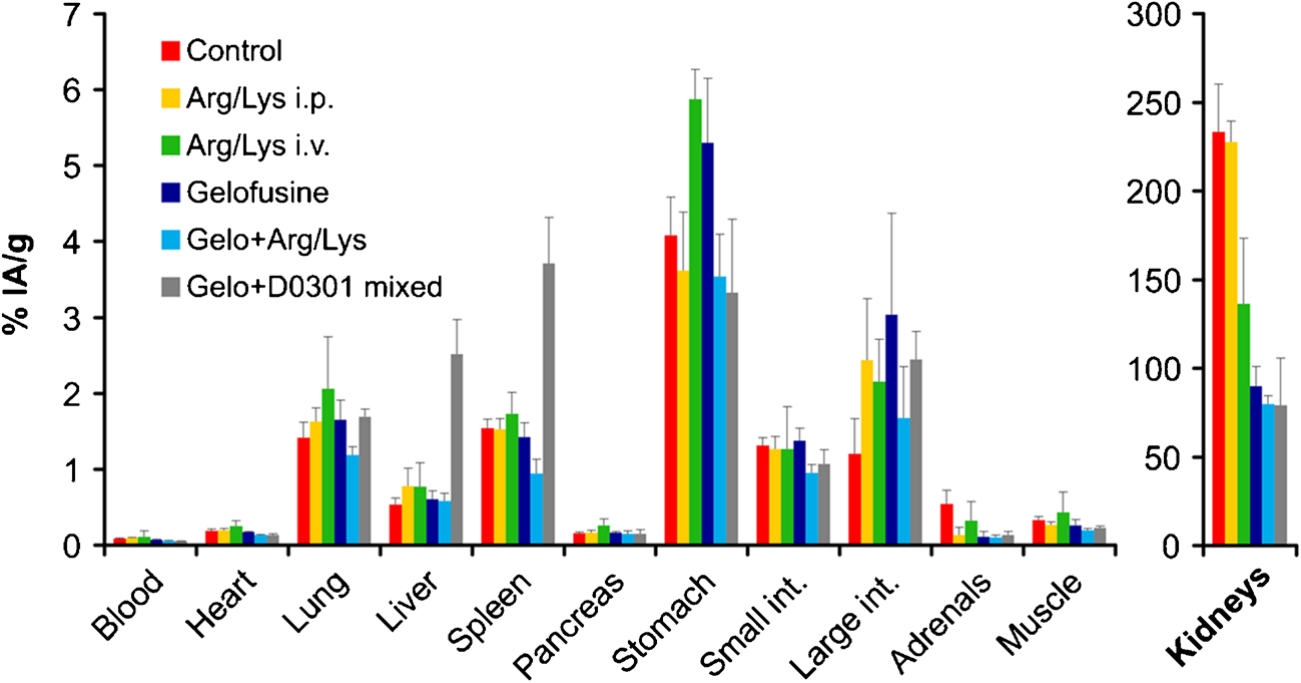
Biodistribution of ^177^Lu-D0301 in C57BL/6 non-tumor bearing mice (24 h p.i.). Control: no kidney protection. Arg/Lys i.p.: 2 × 100 µL Arg/Lys, i.p., 30 min before/after activity. Arg/Lys i.v.: 100 µL Arg/Lys, i.v., 2 min before activity. Gelofusine: 100 µL gelofusine, i.v., 2 min before activity. Gelo + Arg/Lys: 100 µL Arg/Lys + 100 µL gelofusine, i.v., 2 min before activity. Gelo + D0301 mixed: 100 µL gelofusine, mixed with activity. Data are displayed as averages ± SD of *n* = 5 per group

The results of these experiments are summarized in Figs. 2 and  3. We found that a single i.v. injection of 100 µL Arg/Lys immediately (2 min) before the radiopharmaceutical generally reduced kidney uptake more efficiently than two i.p. injections of 100 µL Arg/Lys, 30 min before and after the activity, as compared to the control group that received no renal protection. This pattern was observed for ^68^Ga-Trivehexin, 90 min p.i. (i.v. − 25% vs. i.p. − 9.6%, relative to control; see Fig. 2), as well as for ^177^Lu-D0301, 24 h p.i. (i.v. − 42% vs. i.p. − 2.6%; see Fig. 3). Hence, the i.v. administration route was chosen for all further experiments since it was found to be more effective with a lower overall injected amount of kidney protecting agent.

Intravenous administration of 100 µL gelofusine 2 min before the radiopharmaceutical reduced kidney uptake to a much larger extent than 100 µL of Arg/Lys. The difference between both agents was more pronounced for ^68^Ga-Trivehexin, 90 min p.i. (Gelo − 70% vs. Arg/Lys − 25%, relative to control) than for ^177^Lu-D0301 at 24 h p.i. (Gelo − 61% vs. Arg/Lys − 41%). Since both agents showed a considerable effect for ^177^Lu-D0301, we also tested a combination of gelofusine and Arg/Lys which, however, resulted only in a minor improvement (Gelo + Arg/Lys: − 66%) as compared to gelofusine alone (− 61%).

The formulation of ^177^Lu-D0301 in gelofusine had approximately the same effect on kidney uptake as administration of gelofusine 2 min before the radiopharmaceutical (− 66% vs. − 61%, respectively), but increased the hepatic and splenic uptake by factors of 4.7 and 2.4, respectively (Fig. 3). This unfavorable alteration of the pharmacokinetic profile was presumably caused by aggregate formation, which led us to abandon the approach.

Besides kidneys, Arg/Lys slightly reduced the ^68^Ga-Trivehexin uptake in some organs (lung, spleen, and gastrointestinal tract) (Fig. 2). There was no other systematic variation in any other organ or tissue, and virtually no changes were observed with gelofusine. More pronounced alterations in the general biodistribution were noticed for ^177^Lu-D0301, for example, a considerably higher uptake of ^177^Lu-D0301 in the stomach for all renal protectants. These findings are difficult to interpret, and no rationale can be given at present, particularly in view of the fact that they were not consistently reproduced in another mouse strain (see below). Of course, the limited number of animals per group (*n* = 5) does not always lead to fully representative data because of a usually high intersubject variability, and experimental bias cannot be fully ruled out at all times. The apparently higher intestinal absorption caused by all renal protectants could therefore also be due to a coincidentally lower uptake in the large intestines of all animals of the control group.

### Renal protection agents in tumor-bearing mice

**Fig. 4:**
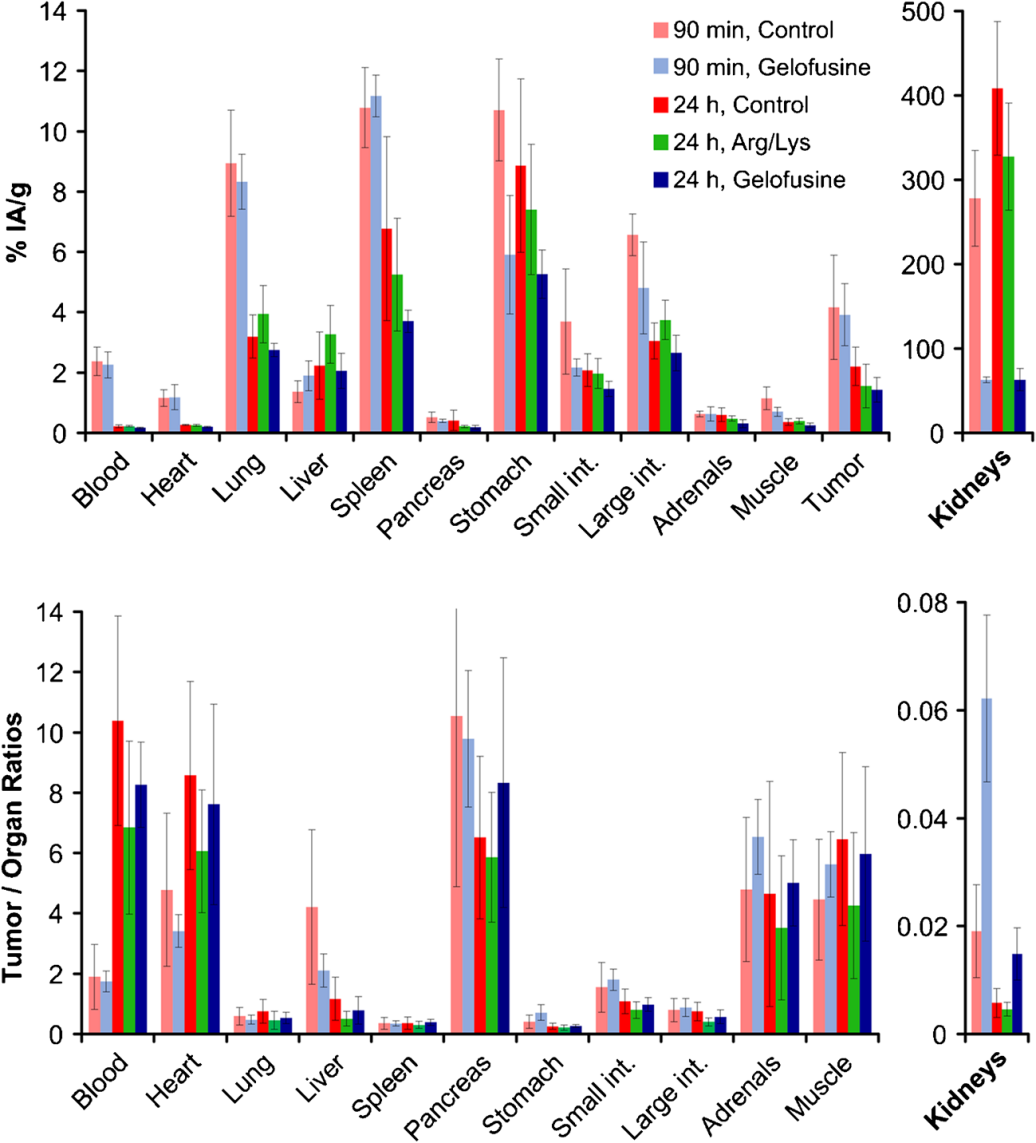
Biodistribution of ^177^Lu-D0301 in H2009 (human lung adenocarcinoma) xenografted SCID mice. Control: no kidney protection. Arg/Lys: 100 µL Arg/Lys, i.v., 2 min before activity. Gelofusine: 100 µL gelofusine, i.v., 2 min before activity. Data are displayed as averages ± SD of *n* = 5 per group

The effect of the most promising kidney protection schemes, i.v. administration of Arg/Lys or gelofusine 2 min before the radiopharmaceutical, was evaluated for ^177^Lu-D0301 in SCID mice xenografted with the αvβ6-integrin expressing human lung adenocarcinoma cell line H2009 for two different time points (90 min and 24 h, see Table 2 and Fig. 4).

**Table 2:** ^177^Lu-D0301 biodistribution experiments performed in H2009-tumor bearing SCID mice (*n* = 5/group)

Time p.i	Kidney protection scheme	Dose [mg/kg]	Peptide dose [pmol]
90 min	none		62 ± 22
	100 µL gelofusine, i.v., 2 min before activity	200	71 ± 22
24 h	none		66 ± 19
	100 µL Arg/Lys, i.v., 2 min before activity	250	66 ± 24
	100 µL gelofusine, i.v., 2 min before activity	200	104 ± 23

For the 90 min time point, only gelofusine was tested. There was no influence on tumor and organ uptakes except a moderate reduction in the gastrointestinal tract (stomach − 45%, intestines − 27%) but, as expected, a much lower kidney activity (− 77%, relative to control). Both protectants reduced the tumor uptake 24 h p.i. (Arg/Lys − 29% and Gelo − 35%), which is considered similar with respect to the error margins. Gelofusine furthermore reduced all organ uptakes to a varying extent, while no consistent pattern was observed for Arg/Lys. Again, the effect on renal uptake was different for both agents, with gelofusine reducing the activity in the kidneys > 4 times more efficiently than Arg/Lys (− 85% vs. − 20%, respectively, relative to control).

The effects of Arg/Lys on tumor and kidney uptake thus compensated each other and the average tumor-to-kidney ratio at 24 h p.i. was not improved by Arg/Lys (Fig. 4). In contrast, gelofusine substantially increased the tumor-to-kidney ratio by factors of 3.3 (90 min p.i.) and 2.6 (24 h p.i.). The other tumor-to-organ ratios remained similar with respect to the error margins for both agents.

### PET and SPECT imaging

**Fig. 5:**
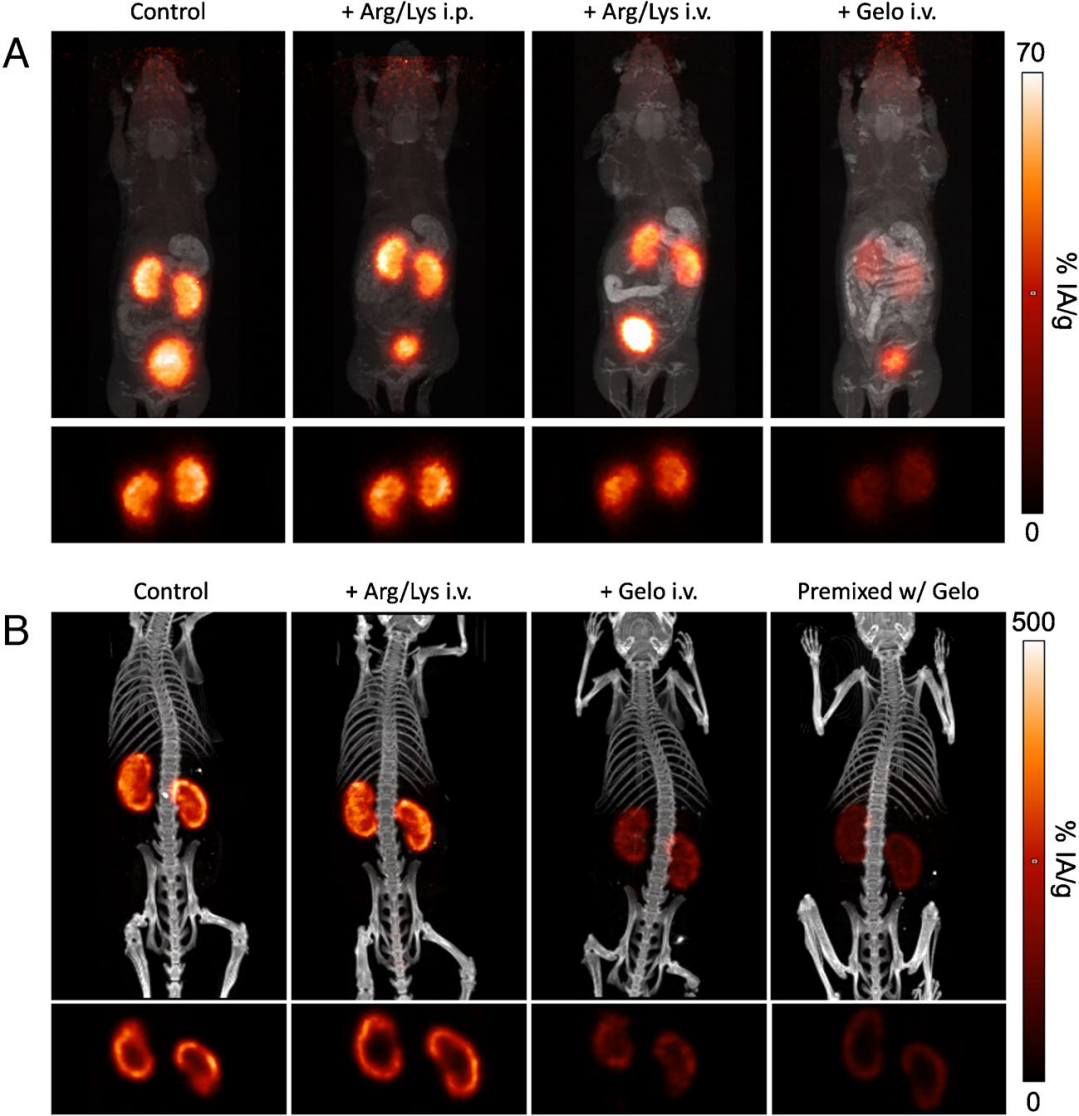
Images of non-tumor bearing C57BL/6 mice without (control) and with kidney protecting agents. **A** ^68^Ga-Trivehexin PET/MRI, 90 min p.i.; **B** ^177^Lu-D0301 SPECT/CT, 24 h p.i.. Large images show fusions of maximum intensity projections (MIPs) for PET & MRI (**A**) and SPECT & CT (**B**). Small images below fusions show respective axial slices through kidneys

The practical implications of reduction of renal uptake of the PET tracer ^68^Ga-Trivehexin were showcased by PET/MR as well as SPECT/CT imaging (Fig. 5). In accordance with biodistribution, a reduced signal intensity was observed for i.v. Arg/Lys and, to a much higher extent, for i.v. gelofusine co-infusion. Axial slices of SPECT images though kidneys demonstrated that the radiopharmaceutical was retained in the kidney cortex (Fig. 5B, bottom row). In the corresponding PET slices, cortical retention could not be directly observed due to the limited resolution of ^68^Ga-PET, resulting from the pronounced positron blurring of this nuclide (Fig. 5A, bottom row).

## Discussion

### Mechanistic considerations

In this study, the administration of succinylated gelatin prior to injection of radiolabeled αvβ6-integrin ligands reduced their renal uptake in two different mouse strains by up to 85%. The observed renal uptake reduction was several times higher than that achieved by infusion of arginine/lysine solutions. This finding could be related to the pharmacokinetics of the renal protection agents. Small molecules such as amino acids are commonly cleared from the plasma much faster than high-molecular weight colloids such as succinylated gelatin. A bolus of gelofusine may therefore have exerted its renal protective effect over a longer period of time than Arg/Lys, which may have resulted in a comparatively more efficient overall blockade.

A review of the literatures makes it also plausible to assume that the difference in efficiency is primarily due to different modes of action of the agents. The pertinent literature mentions a large variety of receptors potentially responsible for the renal uptake and retention of radiolabeled peptides. In this context, the megalin/cubilin transporter system has been studied in detail using knock-out mice, and has been found to be of high relevance for renal uptake of peptides and proteins [3, 26]. The renal protective effect of both gelofusine and Arg/Lys is widely considered to rely on the blockade of protein binding sites of megalin and/or cubilin [27]. Megalin possesses four large extracellular binding domains with different substrate specificities [28]. In this context, Béhé et al. reported that renal uptake of minigastrin and exendin could be reduced by polyglutamic acid (PGA) more efficiently than by cationic amino acids, whereas the opposite was found for radiolabeled octreotide [29]. PGA is structurally similar to succinylated gelatin because both are polymers bearing terminal carboxylic acid moieties in their molecular repeats and thus, belong to the group of polyanionic compounds. It is therefore plausible to assume that the observed selective reduction of renal uptake of our αvβ6-integrin binding peptide multimers by gelofusine was also caused by specific blockade of a certain megalin or cubilin binding site that cannot be blocked by cationic amino acids.

Similar observations have been made for other radiolabeled compounds, which also appear to be more or less selectively recognized by different binding sites of megalin/cubilin, resulting in different blocking efficiencies of the various renal protectants. For example, a dose of 80 mg gelofusine per kg body weight in rats reduced 50–60% of the renal uptake of ^111^In-DOTATATE, which could be improved to 70% by additional Lys infusion [30]. Gelofusine has also been found to be more efficient than Lys in case of ^99m^Tc-labeled nanobodies (approx. 35% reduction vs. 25% for Lys only), and an additive effect was observed as well (approx. 45% reduction for the combination) [26]. A different result was obtained for a radiopharmaceutical whose structure is somewhat related to the compounds in our study, namely, a RAFT-based tetramer of the integrin ligand c(RGDfK) with a ^64^Cu-cyclam label [31]. Lys co-injections showed no effect here, whereas approx. 34% reduction was achieved with gelofusine alone as well as with a combination with Lys. For the same system tagged with ^111^In-DOTA, pre-injection and co-injection of gelofusine reduced the activity in the kidney by 49% and 48%, respectively, while the uptakes in tumor and organs of the used subcutaneous HEK293(β3) xenograft mice were not significantly changed [32]. In contrast, gelofusine had no effect on the renal uptake of different radiometalated CCK2-binding peptidomimetics [33]. Furthermore, both gelofusine and Lys did not affect the renal uptake of the CD38-specific single domain antibody ^68^Ga-NOTA-Nb1053 [34], of engineered protein scaffolds like designed ankyrin repeat proteins (DARPins, typical size 14–18 kDa) [35], and of albumin binding domain derived affinity proteins (ADAPTs, typical size 5 kDa) [36]. An important conclusion from the above considerations is that it is advisable to test different kidney protection systems for new classes of radiopharmaceuticals without making any prior assumptions, as their efficiency can hardly or not at all be predicted on the basis of previous experience. Since preclinical results in rodents have only limited predictive value for humans, this also applies for clinical translation.

### Risks associated with the use of succinylated gelatin in RLT

The risks associated with gelatin-based plasma substitutes were summarized in 2016 in a comprehensive meta-analysis of non-randomized studies and randomized trials comparing this treatment of shock with the infusion of electrolyte solutions [37]. The authors conclude that the use of gelatin infusions is associated with an increased risk of renal failure, bleeding, and death. However, most of the trials and studies discussed were conducted in the context of major surgery, emergencies, intensive care, and severe sepsis. The reported risk figures can therefore not be applied to the use of succinylated gelatin in RLT.

Nonetheless, there is a risk of anaphylactic reactions to the infusion of a foreign protein [37], which deserves attention in this context. An almost 50 years old study put the incidence of anaphylaxis in connection with the application of gelatin-based plasma expanders at 0.038% or 38 per 100,000 applications [38]. In 2018, a year-long audit reported three cases of anaphylaxis due to intravenous gelatin-based solutions from an estimated 52,160 administrations in the UK, resulting in a rate of 6.2 per 100,000 applications (0.0062%) [39]. In a detailed study on a small cohort of 12 patients with known peri-operative hypersensitivity to intravenous succinylated gelatin-based solutions, a median delay of 15 min (range 2 to 70 min) to the onset of partly severe reactions was observed [40]. In this study, gelatin hypersensitivity was confirmed by intradermal tests in 11 patients and by intravenous provocation in one patient with negative skin prick and intradermal tests. Serum tryptase was measured in nine patients and in all nine a significant rise was observed. Interestingly, in another case report of a fatal cardiac arrest caused by a perioperative hypersensitivity reaction following multiple succinylated gelatin infusions, the authors also reported a highly elevated serum tryptase level [41]. Such findings and figures need to be taken into account to develop suitable risk management strategies.

Taken together, there is a low but not negligible risk of anaphylaxis associated with gelofusine infusions (0.0062–0.038%) [38, 39], requiring a suitable risk management strategy. To put this into perspective, it should be noted that the estimated rates of severe adverse reactions (including but not limited to anaphylaxis) during the application of iodinated or gadolinium-based contrast agents are 0.04% [42] and 0.008% [43], respectively, an incidence that has been referred to as ‘uncommon’ [44]. Nonetheless, appropriate patient supervision and emergency protocols are a part of the clinical routine for contrast-enhanced imaging procedures [44]. These could be adapted for succinylated gelatin infusions during RLT.

## Conclusion

In view of the clinical prospects of the αvβ6-integrin targeting peptide trimer ^68^Ga-Trivehexin for PET imaging of various carcinomas, a corresponding therapeutic radiopharmaceutical for RLT is highly desirable. The first generation of respective ^177^Lu-labeled peptide tetramers however showed a high renal uptake, which diminished their prospects for translation into the clinics due to potential renal toxicity. Co-infusion of succinylated gelatin led to a strong reduction of renal uptake of up to 85% in tumor-bearing mice, suggesting it as the kidney protection agent of choice for this compound class. Due to the known low but not negligible incidence of side effects with gelofusine infusions, appropriate risk management strategies, comparable to those applied for the use of CT or MRI contrast agents, are required in case of future clinical use. Finally, with the identification of a potent renal protection strategy, our development of αvβ6-integrin targeted radiotherapeutics has nonetheless not yet reached its final stage, as improved tumor uptake and retention as well as inherently lower renal absorption are required for clinical success [1].

## Data Availability

The datasets used and/or analysed during the current study are available from the corresponding author on reasonable request.
